# Arginase as a Potential Biomarker of Disease Progression: A Molecular Imaging Perspective

**DOI:** 10.3390/ijms21155291

**Published:** 2020-07-25

**Authors:** Gonçalo S. Clemente, Aren van Waarde, Inês F. Antunes, Alexander Dömling, Philip H. Elsinga

**Affiliations:** 1 Department of Nuclear Medicine and Molecular Imaging, University Medical Center Groningen, University of Groningen, 9713 GZ Groningen, The Netherlands; g.dos.santos.clemente@umcg.nl (G.S.C.); a.van.waarde@umcg.nl (A.v.W.); i.farinha.antunes@umcg.nl (I.F.A.); 2 Department of Drug Design, Groningen Research Institute of Pharmacy, University of Groningen, 9713 AV Groningen, The Netherlands; a.s.s.domling@rug.nl

**Keywords:** arginase, nitric oxide, arginase inhibitors, molecular imaging, positron emission tomography (PET)

## Abstract

Arginase is a widely known enzyme of the urea cycle that catalyzes the hydrolysis of L-arginine to L-ornithine and urea. The action of arginase goes beyond the boundaries of hepatic ureogenic function, being widespread through most tissues. Two arginase isoforms coexist, the type I (Arg1) predominantly expressed in the liver and the type II (Arg2) expressed throughout extrahepatic tissues. By producing L-ornithine while competing with nitric oxide synthase (NOS) for the same substrate (L-arginine), arginase can influence the endogenous levels of polyamines, proline, and NO^•^. Several pathophysiological processes may deregulate arginase/NOS balance, disturbing the homeostasis and functionality of the organism. Upregulated arginase expression is associated with several pathological processes that can range from cardiovascular, immune-mediated, and tumorigenic conditions to neurodegenerative disorders. Thus, arginase is a potential biomarker of disease progression and severity and has recently been the subject of research studies regarding the therapeutic efficacy of arginase inhibitors. This review gives a comprehensive overview of the pathophysiological role of arginase and the current state of development of arginase inhibitors, discussing the potential of arginase as a molecular imaging biomarker and stimulating the development of novel specific and high-affinity arginase imaging probes.

## 1. Introduction

**Figure 1:**
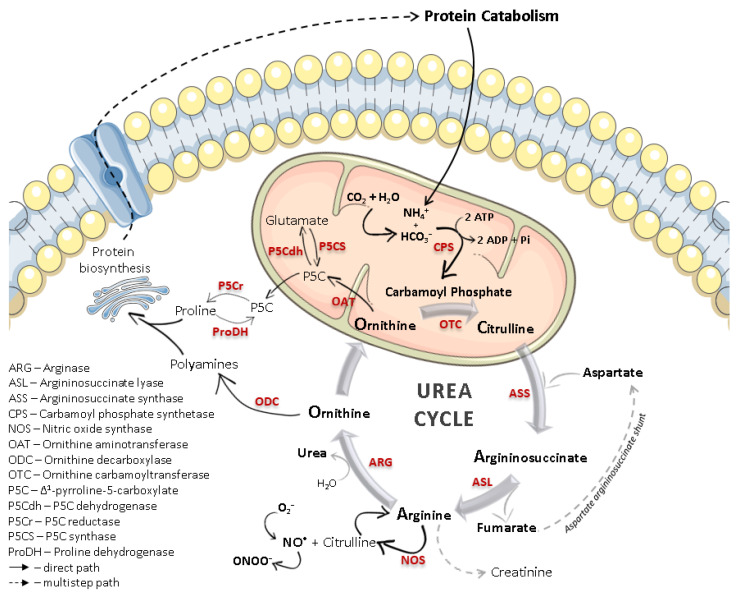
Scheme of the urea cycle, including the overall role of L-ornithine within (highly restricted to recycling) and outside of this cycle (regulation of protein synthesis). The competitive L-arginine metabolism between arginase and nitric oxide synthase (NOS), occurring in many non-hepatic cell types, is also represented. Not all the outlined processes occur in every cell type, and their expression and extent may depend on several physiological or pathological processes.

The identification of the Krebs–Henseleit urea cycle in the early 1930s highlighted the importance of arginase, a manganese-containing enzyme that catalyzes the conversion of L-arginine to urea and L-ornithine. The individual hydrolytic function of arginase was previously known, but the interdependence of arginase and other biochemical mechanisms, as evidenced in the urea cycle (Figure 1), triggered scientific interest. Despite the early findings showing that arginase was mostly expressed in the mammalian liver [1], and to a lesser extent in kidneys [2], this enzyme was also identified in organs where the urea cycle is not present [3,4,5]. Thus, investigations on the L-arginine metabolism in non-ureagenic tissues revealed a parallel role of arginase beyond ureagenesis: to regulate L-ornithine levels and subsequent polyamine and proline biosynthesis.

With the isolation of arginase from different rodent tissues, and comparing the physicochemical properties, it became evident that different isoforms exist. Extending these studies to human tissues led to similar results, and the co-existence of two arginase isoforms became widely accepted by the scientific community since the early 1980s [6]. Due to the abundance in subcellular compartments, arginase type I (predominantly expressed, but not exclusively, in the liver and with a prominent role in the urea cycle) became described as cytosolic, and type II (widely expressed in extrahepatic tissues and mainly involved in the production of L-ornithine outside the urea cycle) as mitochondrial.

### 1.1. Arginase Isoforms

In mammals, Arg1 genes are mostly expressed in the liver, and to a much lesser extent in bone marrow, whereas Arg2 genes are present in virtually all tissues (with predominance in the kidney, prostate, digestive and gastrointestinal tract, muscle, and endocrine tissues) [7]. Despite keeping the same enzymatic function, the different biochemical context in these tissues favors two complementary roles. Hepatic cytosolic Arg1 plays a primary role in the net production of urea (for ammonia clearance) and in the biosynthesis of L-ornithine, which is usually recycled within the urea cycle. Mitochondrial Arg2 mainly regulates the physiological biosynthesis of L-ornithine in several other tissues. L-Ornithine is a precursor of polyamines (putrescine, spermidine, spermine), proline, and glutamate, which are essential for collagen synthesis, tissue repair, cell proliferation, growth and viability, and neuronal development, and in the regulation of immune and inflammatory responses [8].

**Figure 2:**
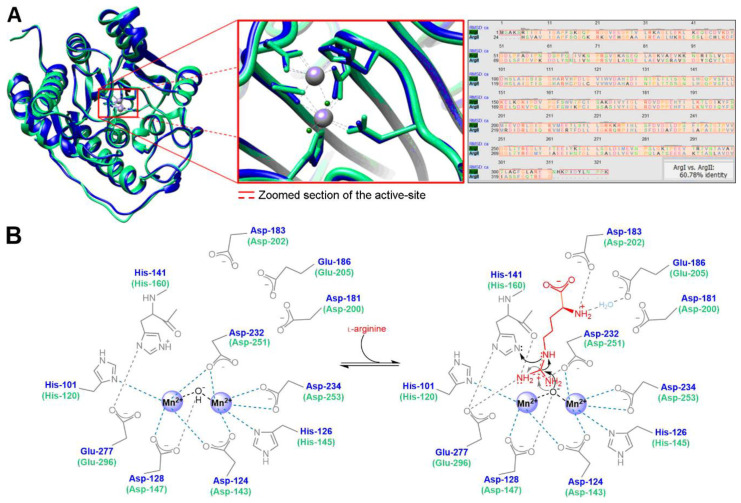
(**A**) Superposition of human Arg1 and Arg2 (blue and green color, respectively) subunits and active-sites and amino acid sequence alignment showing the shared homology percentage by both isoforms (molecular graphics and analyses performed with UCSF Chimera [12] using PDB accession codes 2ZAV [13] and 1PQ3 [9]). (**B**) Schematic overview of the most relevant active-site amino acid residues involved in catalytic plasticity, and proposed L-arginine binding mechanism.

High-resolution crystallography techniques allowed characterizing the structure of human arginase and detailing the differences between the isoforms. Human Arg1 (105 kDa) and Arg2 (129 kDa) exist primarily as homotrimeric metalloenzymes encoding 322 and 354 amino acid residues, respectively [9,10]. Despite being encoded by different genes, approximately 61% of the amino acid sequence identity is shared by both isoforms, and all active-site residues involved in substrate binding, as well as the binuclear Mn^2+^ cluster core, are strictly conserved (Figure 2A). These Mn^2+^ ions are approximately 3.3 Å apart, bridged by an OH^−^ ion, and mainly surrounded by negatively charged amino acid residues (Figure 2B), which form an electron paramagnetic resonance spin-coupled binuclear center located at the bottom of a 15 Å deep cleft in each of the three identical subunits [11].

Since the active-site residues of type I and type II arginase are identical, and both enzymes require the binuclear Mn^2+^ cluster core, the overall mechanism of L-arginine hydrolysis is thought to be similar. Due to the specific arrangement of the hydrogen bond donor residues at the active-site, both arginase isoforms are highly specific in recognizing and binding amino acids containing α-amino and α-carboxylate groups and are also stereochemically selective. The side chain of the Glu-277 (or Glu-296 for Arg2) residue forms a salt bridge with the scissile guanidinium carbon from L-arginine, which seems essential to the recognition, alignment, and directing of the substrate to the metal-bridging hydroxide for further nucleophilic attack. L-Arginine is additionally stabilized at the optimal conformation by hydrogen bonds between its α-amino group and Asp-183/Glu-186 (or Asp-202/Glu-205), making the length of the carbon chain from the ligand crucial for the catalytic activity [14]. The nucleophilic attack to the guanidinium carbon produces a tetrahedral intermediate stabilized by the binuclear Mn^2+^ center. This intermediate then collapses to produce L-ornithine and the by-product urea. In parallel, the side-chain of His-141 (or His-160), stabilized by a hydrogen bond with Glu-277 (or Glu-296), is involved in the shuttle of protons from the bulk solvent to the active-site, allowing the complete dissociation of the produced L-ornithine [15].

Despite the active-site residues and the proposed catalytic mechanisms being the same for both arginase types, the kinetic behavior of the isoforms shows some differences (*K_m_* Arg1 = 3.3 mm and *K_m_* Arg2 = 1.9 mm at pH 7.4; *V_max_* Arg1 = 34 nmol.min^−1^.mg^−1^ and *V_max_* Arg2 = 0.9 nmol.min^−1^.mg^−1^ [16]). Each subunit of the trimer follows an α/β fold structure comprising a central parallel-eight-stranded β-sheet flanked on both sides by several α-helices [17,18], which, due to some length and amino acid sequence differences of the isoforms, translates into minor structural variations at the active-site (Figure 2A). These differences marginally change the bond lengths between the ligand and each isoform active-site. Consequently, there are variances in the isozyme-ligand kinetics, as well as different sensitivity and responsiveness of each arginase subtype toward potential inhibitors.

### 1.2. Arginase/Nitric Oxide Synthase (Patho)Physiological Interplay

Variations in arginase levels are known to cause changes in L-arginine bioavailability and, consequently, an imbalance in the production of L-ornithine and its downstream metabolites (polyamines and proline), triggering the deregulation of protein synthesis, which may lead to multiple systemic abnormalities (e.g., fibrosis, cell proliferation) [19]. Changes in nitric oxide (NO^•^) levels are also associated with these situations since L-arginine (when outside the urea cycle) is simultaneously the only physiological substrate for nitric oxide synthase (NOS), an enzyme that exists in endothelial (eNOS), neuronal (nNOS), and inducible (iNOS) isoforms, and catalyzes the production of NO^•^ and the by-product L-citrulline (Figure 1). Despite NOS having a higher affinity for L-arginine (*K_m_* e/nNOS ≈ 2.2 µm at pH 7.4 [20,21] and *K_m_* iNOS = 16.0 µm at pH 7.5 [22]), arginase has 10^3^–10^4^ times higher *V_max_* [23]. The superior reaction velocity (*V_max_*/*K_m_*) of arginase makes it a very effective competitor of NOS [24]. Thus, an upsurge of arginase can deprive NOS of its substrate, fading NO^•^ signaling levels and the associated physiological effects (e.g., modulation of vascular and airway tone, and regulation of the neuronal development and immune response) [25]. Eventually, an extreme depletion of L-arginine may cause NOS uncoupling, with superoxide anion (O_2_^•−^) being favorably produced over NO^•^ and promptly reacting with NO^•^ to form cytotoxic peroxynitrite species (ONOO^−^) [26].

The reactive and diffusion properties of NO^•^ make this gas a crucial cellular signaling molecule capable of regulating many biological processes. Most cells can produce NO^•^ via expression of one or more isoforms of NOS. Endothelial and neuronal NOS, named after the tissues in which they were first identified, are generally constitutively expressed and, upon phosphorylation in specific tyrosine residues by Ca^2+^/calmodulin-dependent kinases, produce NO^•^ [27]. The physiological endothelial release of NO^•^ activates the phosphorylation of several signaling proteins. These signals may stimulate, for example, the production of vascular endothelial growth factor and the relaxation mechanisms of smooth muscle [28]. Beyond controlling the vascular tone by modulating smooth muscle cell proliferation, NO^•^ also has antithrombotic effects by inhibiting platelet aggregation and preventing leukocyte adherence to the endothelium, being a unique signaling molecule in several physiological mechanisms of cardiovascular protection [29]. Similarly, physiological levels of NO^•^ produced by nNOS in the nervous system stimulate synaptic plasticity and neuronal modulation [30]. In contrast, iNOS is highly expressed in macrophages and can be activated in a Ca^2+^-independent manner by several immunoinflammatory stimuli, such as nicotinamide adenine dinucleotide phosphate oxidase 2 (NOX2), interleukin-1 (IL-1) family, tumor necrosis factor (TNF), or γ-interferon (IFN-γ) [31,32,33,34]. The NO^•^ produced by iNOS mainly acts by modifying the redox profile of the target microenvironment to enable non-specific immune-defense mechanisms for the eradication of pathogens [35]. However, if deregulated, these effects may also evoke oxidative stress and cytotoxicity in non-harmful cells.

Similar to what happens with iNOS, and especially in cells of the immune system, arginase expression and activity can also be modulated by immune regulatory signals, such as several interleukins (IL) or the transforming growth factor β (TGF-β) [36]. Therefore, the overall biochemical context is capable of influencing the arginase/NOS balance, and each enzyme can reciprocally up- or downregulate the activity of the other through L-arginine depletion and counter-regulatory mechanisms. Beyond substrate competition, there are multiple cross-inhibitory interactions between both L-arginine metabolic pathways. For example, even if deprived of L-arginine, NOS can self-regulate NO^•^ synthesis by recycling the by-product L-citrulline back to L-arginine in the presence of argininosuccinate synthase and lyase (Figure 1). NO^•^ was also reported to inhibit ornithine decarboxylase, the enzyme responsible for the catalysis of L-ornithine to polyamines, which may indirectly mitigate the effects of an increase in arginase activity [37]. On the other hand, certain polyamines are equally capable of suppressing NOS activity [38]. Furthermore, an intermediate in NO^•^ biosynthesis, *N*^ω^-hydroxy-L-arginine (NOHA), was found to exert an inhibitory effect towards arginase, increasing L-arginine availability for NO^•^ production [39].

**Figure 3:**
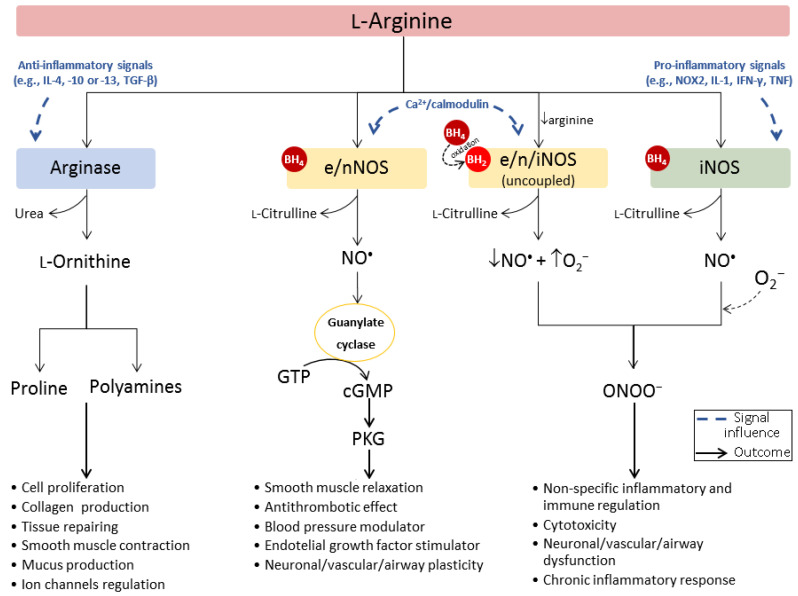
Scheme of competitive L-arginine metabolism via arginase (outside urea cycle) and NOS. The biochemical context influences the L-arginine metabolic pathway taken, the balance, and extent of the final products, inducing a more protective or pathological outcome.

Depending on the specific biochemical context, the NO^•^/L-ornithine imbalance may lead to protective or harmful consequences to the target cells or tissues, culminating in physiological or pathological processes (Figure 3). Thus, understanding of the main regulatory mechanisms adjusting to (as a response to pathological or deregulatory mechanisms), or taking advantage of (e.g., by some pathogens), this arginase/NOS dichotomous outcome, is crucial for the development of successful therapeutic or diagnostic strategies.

## 2. The Pathophysiological Role of Arginase

The co-expression of two arginase isoforms and three NOS isoforms, together with a series of induction factors and feedback loops, make the L-arginine metabolic pathway a remarkably complex signaling cascade. The homeostasis of all these signals is crucial to keep the functionality of the organism. When expressed together with NOS, arginase can regulate NO^•^, polyamines, and proline production, especially in the immune system [8], endothelial [40], and neuronal cells [41]. A series of vascular, neuronal, immune, and inflammatory pathologies may then arise from the disturbance of arginase expression and activity. Therefore, the arginase expression and L-arginine bioavailability levels are potential biomarkers of disease progression and severity.

### 2.1. Immune System Cells

Generally, in the absence of inflammatory stimuli, macrophages produce low levels of NO^•^ and remain in a dormant state. However, after activation with a stimulus such as lipopolysaccharide-induced inflammation, TNF, or IFN-γ, the NO^•^ levels increase significantly and continuously as long as an adequate extracellular concentration of L-arginine is still present [42,43,44]. The NO^•^ synthesized by iNOS in immune cells can interact with reactive oxygen species to induce a cytotoxic nitrosative stress environment, which inhibits pathogenic replication and activity [35]. In the course of an inflammatory process, macrophages can switch between a phenotype that expresses arginase or NOS, which changes the L-arginine metabolism outcome [45]. Certain interleukins, cyclooxygenase-2 (COX-2), or TGF-β, can induce macrophages to express arginase, promoting the upsurge of L-ornithine and its downstream metabolites by competition with iNOS for L-arginine pools [46,47,48]. Through the restraint of L-arginine availability, arginase can potentially regulate L-arginine-dependent immune defense mechanisms. For example, Arg2 can downregulate NO^•^ levels, preventing uncontrolled cellular apoptosis triggered by the ONOO^−^ species produced after an excess of NO^•^ reacting with superoxide radicals (O_2_^•−^) [49].

The differences in L-arginine metabolism and chemokine receptor profiles gave rise to a simplified and dichotomous classification for the macrophage phenotypes: M1 catalyzes L-arginine mainly via NOS/NO^•^-L-citrulline, and M2 favors the arginase/L-ornithine metabolic pathway [50]. As opposed to M1, where the innate primary immune response is mediated by activation of pro-inflammatory or antitumor cytokines (type 1 helper T cells) stimulating the apoptosis of pathogens (e.g., antibacterial, antiviral, and antifungal effect), M2 macrophages are involved in restoring mechanisms, debris scavenging, cell proliferation, angiogenesis, antibody formation, and the induction of anti-inflammatory or immune-regulatory cytokines (type 2 helper T cells) [23].

Alongside the importance of a balanced M1/M2 polarization sequence, to maintain homeostasis, and of a regulatory feedback pathway (e.g., type 1 helper T cells stimulate NO^•^ levels but can also be inhibited by this gas to prevent an exacerbated immune response), there is also a microenvironment-dependent multifactorial signaling cascade able to regulate macrophage plasticity and to promote the development and differentiation of T cells and cytokines. Impaired M1/M2 polarization may result, for example, in non-resolving inflammations, autoimmune diseases, allergic conditions, pathogen infections, or neoplastic stages. The deregulated release of arginase from cells and tissues into extracellular fluids may also disrupt macrophage defense mechanisms against pathogens, as it limits L-arginine bioavailability, decreases NO^•^ production, and disturbs the cytokine production pathways [51]. Thus, the mapping of arginase expression holds high potential as a molecular imaging biomarker for the identification and follow up of neoplastic, inflammatory, and allergic disorders.

### 2.2. Cardiovascular Endothelium

Beyond promoting tissue repair and healing, arginase, especially the type II isoform, may either play an essential role in the maintenance of cardiovascular equilibrium or be involved in some of the physiological aging mechanisms that lead to dysfunction [25]. Arginase can equilibrate the NO^•^ levels to reduce endothelial oxidative damaging events while still promoting vasodilation and inhibiting leukocyte and platelet adherence and aggregation. A disturbed balance between L-arginine-degrading enzymes may then account for a wide range of age-related cardiovascular complications such as vascular stiffness, ventricular hypertrophy, hypertension, inflammation, and dysfunction by oxidative stress. Curiously, one of the ways that arginase was found to be upregulated and associated with cardiovascular complications resulted from a high fat/cholesterol diet causing liver damage in mice, which led to the systemic release of the hepatic cytosolic arginase and consequent reduction of circulating L-arginine levels and NO^•^-mediated cardioprotective effects [52]. In the opposite direction, glucose fasting was shown to induce Arg2, which suppresses specific signaling mechanisms and protects hepatocytes from the accumulation of fat, inflammatory responses, insulin resistance, and glucose intolerance [53]. These findings exemplify the impact of arginase in the signaling cascade of diverse physiological homeostatic mechanisms. Therefore, the detection of variations in arginase expression may be explored as a potential molecular imaging strategy to follow and predict cardiovascular disease progression and evaluate endothelial function.

### 2.3. Neuronal Cells

Arginase and NOS are prevalent throughout both peripheral and central nervous systems. The interplay between these enzymes is hampered by the particularly complex cellular composition of the brain. In general, arginase is indispensable for the detoxification of ammonia from the central nervous system and for regulating the biosynthesis of polyamines, essential for neuronal growth, development, and regeneration. In parallel, NO^•^ has a well-established role as a neurotransmitter and is involved in synaptic plasticity and regulation of cerebral blood flow [41]. During the early stages of development, neurons are expected to have high endogenous levels of cyclic adenosine monophosphates (cAMP), which upregulate Arg1 and enhance the synthesis of polyamines, essential for neuronal expansion and survival [54]. With time, and depending on other environmental signals, this expression starts fading to favor the NO^•^-induced cellular plasticity, vascular tone, and neurotransmission. However, as a result of aging, an eventual arginase/NOS imbalance may disrupt NO^•^ production and contribute to neurodegenerative processes [55]. The accumulation of arginase has been reported at sites of β-amyloid deposition, which is associated with L-arginine deprivation and neurodegenerative processes, and may be an attractive molecular imaging target for the evaluation of Alzheimer’s disease progression. In different circumstances, an excessive concentration of NO^•^, mediated by an increased Ca^2+^ influx or by numerous pathophysiological transcription factors suppressing arginase, may lead to neuronal cell death and brain trauma due to the emergence of ONOO^−^ species (excitotoxicity) and a deficient regulation of the blood flow.

### 2.4. Overview of the Pathologies Related to Arginase Deregulation

L-Arginine metabolism is essential for healing and maintaining healthy states, for example, by activating the immune system or by modulating smooth muscle tone and neuroplasticity. However, several physiological, pathological, or pharmacological input signals may disturb the metabolism of L-arginine, usually by up- or downregulating arginase expression and activity, which may lead to several complications such as chronic inflammations, cardiovascular, neurovascular, and neurodegenerative diseases or tumors. Thus, arginase generally acts as a dichotomous factor that may cause different outcomes depending on the surrounding biochemical context and can be either the cause of pathological processes or a response mechanism to achieve homeostasis [56,57].

Table 1 summarizes the most relevant findings from the last five years that associate arginase expression and activity with prevalent pathological conditions.

## 3. Development of Arginase Inhibitors

The majority of the pathologies related to arginase deregulation are connected to the upregulation of at least one of the arginase subtypes and consequent NO^•^ reduction. Thus, the inhibition of arginase is currently gaining special attention as a therapeutic approach, and the development of arginase inhibitors is actually a very active field in medicinal chemistry [57,108].

### 3.1. Arginase Inhibitors from the First and Second Generation

Most α-amino acids of the naturally occurring L-form have some sort of inhibitory effect on arginase, the monoamino acids being predominantly non-competitive inhibitors, whereas the diamino acids are usually competitive inhibitors [109]. Although the integrity of the guanidinium group of the substrate is essential for the arginase inhibitory activity and for an improved ability to cross cell membranes [110], its resemblance to the N-C-N sequence within heterocycles makes some pyridine- and purine-containing amino acids also have a non-competitive or competitive inhibitory activity, respectively [111]. The first synthetic arginase inhibitors were L-ornithine and L-lysine derivatives containing an iodoacetamide motif in the N-terminus to enable the formation of covalent bonds with the sulfide or hydroxyl groups of some amino acid side chains at the active-site [112,113,114]. The most suitable side chain size from L-ornithine led to further investigations using α-difluoromethylornithine, a known irreversible inhibitor of ornithine decarboxylase [115,116], which showed moderate inhibitory constant (*K_i_* = 3.9 mm [117]) for arginase in human colon carcinoma cells.

The isolation of NOHA, an intermediate in NO^•^ biosynthesis with an *N*-hydroxyguanidine moiety replacing the guanidinium group of L-arginine and with potent arginase inhibitory constant (*K_i_* = 42.0 µm [118]), boosted the development of new *N*^ω^-hydroxyamino-α-amino acid derivatives to enhance the specificity for arginase over NOS [119]. An interesting finding on NOHA was its 10 to 18 times improved potency to inhibit arginase in hepatic tissues over non-hepatic tissues [120].

**Figure 4:**
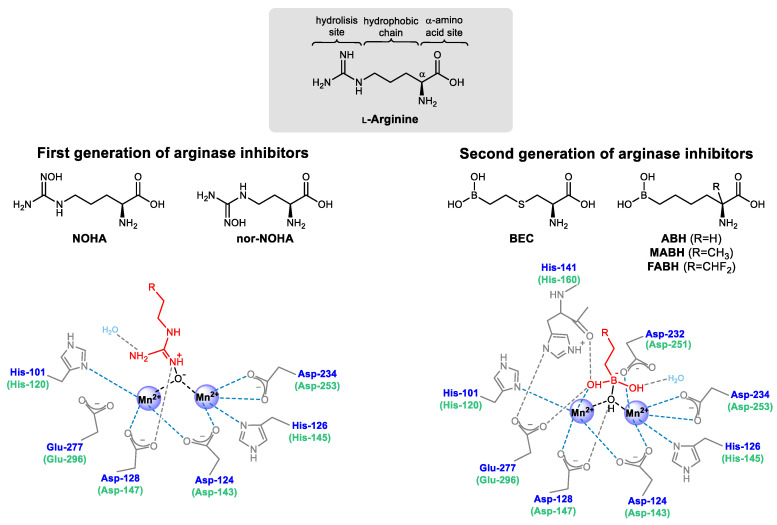
Chemical structure of the most relevant examples from the first and second generation of arginase inhibitors and schematic overview of the binding structure with the binuclear Mn^2+^ cluster.

The one CH*_2_* shorter-chained NOHA derivative, nor-NOHA, which is not a NOS substrate or inhibitor [121,122], became the most potent molecule (*K_i_* = 0.5 µm [123]) of the first generation of competitive reversible arginase inhibitors (Figure 4). The inhibitory activity difference between NOHA and nor-NOHA highlighted the importance of the chain length between the α-amino acid and the hydroxyguanidine function for the recognition by arginase and for further specific interaction with the binuclear Mn^2+^ cluster of the active-site, conferring selectivity to arginase over NOS [119,124]. Pharmacokinetics of nor-NOHA was evaluated in rat models, showing short in vivo target residence time (τ = 12.5 min [125]) and rapid clearance from the plasma (elimination half-life approx. 30 min [126]). Despite the short half-life, the potential of nor-NOHA to inhibit arginase was successfully evaluated in several tumors [127,128], airway [129,130], and cardiovascular [131,132,133,134] disease models. Other L-arginine-like molecules containing guanidine derivatives showed a lack of specificity to arginase and also some inhibitory effect on NOS [135], which would hamper potential therapeutic applications directed to the regulation of L-ornithine/NO^•^ levels.

The finding that simple tetrahedral borate anions have non-competitive inhibitory activity for arginase (*K_i_* = 1.0 mm [136]) and the early characterization of the crystal structure of rat liver arginase [11] allowed understanding better the binding interactions and the transition states with L-arginine during the catalytic activity. Based on these outcomes, 2-(*S*)-amino-6-boronohexanoic acid (ABH, *K_i_* ≈ 0.1 µm [120,137]) was developed. ABH is a slow-binding competitive reversible inhibitor that is rapidly recognized by arginase active-site, which then undergoes slow conformational changes to yield the inhibitory complex [138]. In this second generation (Figure 4) of slow-binding competitive reversible arginase inhibitors, the chemically and metabolically unstable *N*-hydroxyguanidine group is replaced by a boronic acid (B(OH)_2_). The absence of the guanidinium function confers selectivity to inhibit arginase without affecting NOS activity, as the terminal guanidine N-atom is the precursor for NO^•^. When interacting with the binuclear Mn^2+^ cluster, boronic acids are capable of forming structural analogs of the tetrahedral intermediate found during L-arginine hydrolysis (Figure 2B). Unlike the first generation of inhibitors, which bind to arginase by merely displacing the Mn(II)-bridging hydroxide ion [139], the second generation of ligands have one of the boronic acid hydroxyl groups forming a water-mediated bond with threonine, the other OH^−^ directly binding to one of the Mn^2+^ and forming additional hydrogen bonds with histidine (His-141/160) and glutamate (Glu-277/296), while the boronic center displaces and binds to the OH^−^ bridging both Mn^2+^ [140,141]. These additional bonding boosted the inhibitory activity of the second-generation arginase inhibitors.

The therapeutic potential of ABH has been extensively evaluated in several disease models, such as sexual [140,142], immune [83,143], cardiovascular [144,145,146,147], and airway [148,149,150,151] dysfunctions. With improved pharmacokinetics (half-life approx. 8 h [152]) in relation to the first generation of arginase inhibitors, ABH became a reference for the synthesis of further arginase inhibitors. Changes in the side chain size of ABH or the absence of an intact α-amino acid function invariably result in weaker inhibitory activities, whereas the introduction of an alkene functional group in the side-chain limits the flexibility of the arginase inhibitor to fit in the active-site properly [153]. The substitution of the boronic acid function by sulfonamide [154], aldehyde [155], aminoimidazole [156], silanediol [157], nitro, or carboxylic acid groups [158] also generally worsens the inhibitory constant.

The replacement of the central carbon from ABH chain with a sulfur atom yielded *S*-(2-boronoethyl)-L-cysteine (BEC, *K_i_* = 0.5 µm [159]), a compound that has also been used to prove the physiological interdependence between both L-arginine metabolic pathways by experimentally enhancing the NO^•^ levels through the inhibition of arginase [69,96,160,161,162,163,164]. Changes in the side chain size or rigidity of BEC was shown, once again, to negatively affect the inhibitory potency [165].

The first versions of Cα-substituted ABH analogs, 2-amino-6-borono-2-methylhexanoic acid (MABH, *K_i_* ≈ 0.5 µm [166]) and 2-amino-6-borono-2-(difluoromethyl)hexanoic acid (FABH, *K_i_* ≈ 17.0 µm [166]) revealed new regions within the arginase active-site with the potential to form additional hydrogen bonds without compromising the recognition of the ligand.

In summary, some structural conditions seem to be essential for the modulation of inhibitory potency: (a) a side chain length and hydrophobicity similar to L-arginine, to enhance the recognition by arginase; (b) an electron-deficient boronic acid moiety for the displacement of the Mn(II)-bridging hydroxide ion and additional binding to one of the Mn^2+^ ions, to mimic the tetrahedral intermediate produced in the hydrolysis of L-arginine and to confer specificity for arginase over NOS; (c) an α-amino acid group for the conservation of an array of hydrogen bonds with the surrounding amino acid residues from the active-site, to stabilize the inhibitor in the appropriate conformation; and (d) a substituent at Cα with nature, stereochemistry, and bulkiness not disturbing (or, ideally, improving) the hydrogen bonding network and the enzymatic recognition. Substitutions at Cα seem to keep the pharmacophore intact and to affect the molecular recognition minimally. Therefore, Cα is the ideal position to potentially introduce an imaging entity, such as a radionuclide, fluorophore, or a paramagnetic metal ion.

### 3.2. Third Generation of Arginase Inhibitors

**Figure 5:**
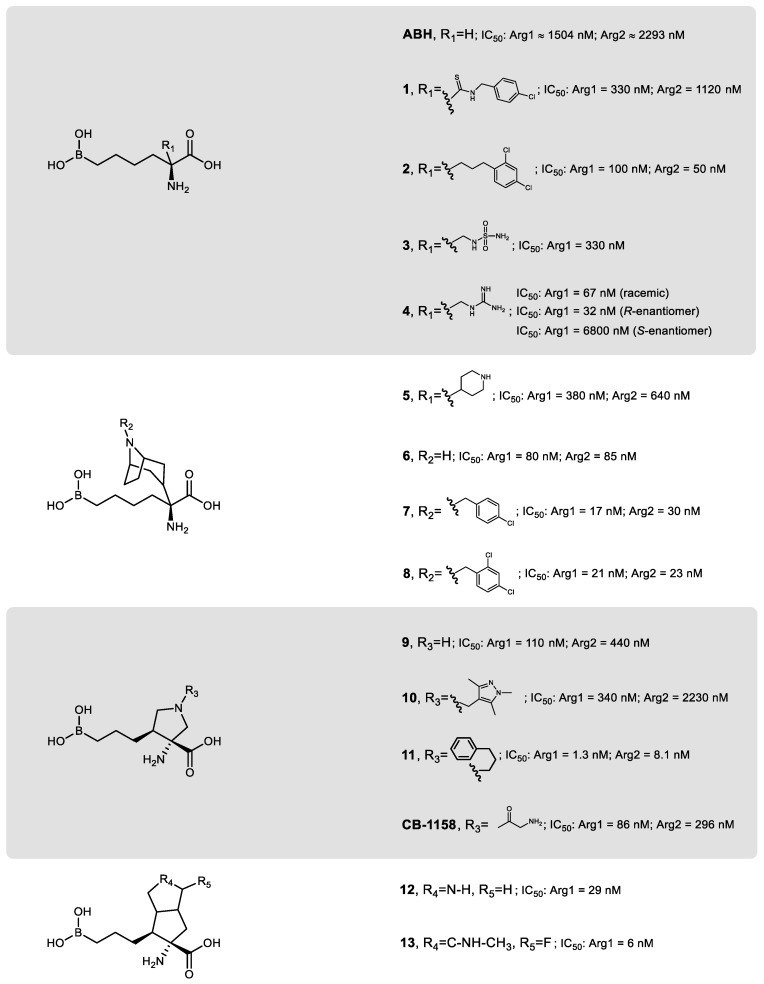
Chemical structure of ABH and some of the most relevant scaffolds from the ABH-based Cα substituted generation of arginase inhibitors.

The design of isoform-specific arginase inhibitors has shown to be challenging since the difference between the active-site of Arg1 and Arg2 is limited to minor structural variations (Figure 2A). To try to tackle this issue, taking advantage of the enzyme plasticity [167], and since MABH and FABH showed additional interactions with peripheral regions of the arginase active-site without compromising the inhibitory potency [166], a third generation of arginase inhibitors based on Cα-substituted ABH analogs arose (Figure 5).

To have a faster screening capacity of the libraries of arginase inhibitors synthesized, the measuring of the half-maximal inhibitory concentration (IC_50_) became more predominant than *K_i_*. Even though *K_i_* allows comparing values between different laboratories, as it is independent of enzyme and substrate concentrations, IC_50_ enables faster measurements and speeds up the determination of the relative inhibitory potency, as fewer data points are required [168].

Although substitutions at the Cα of ABH analogs represent a successful strategy to decrease the IC_50_ value, structure–activity relationship analysis of these inhibitors also confirmed the difficulty of achieving a significant selectivity between Arg1 and Arg2 [169,170,171]. Thus, arginase inhibitors with pharmacologically significant selectivity to one of the isoforms over the other are still not available. The most considerable difference between isozymes IC_50_, favoring Arg1 over Arg2 in about 3.4 times, was initially achieved for the *N*-(4-chlorophenyl)ethanethioamide-substituted ligand (**1**) [171] (Figure 5). On the other hand, the 1-butyl-2,4-dichlorophenyl-substituted ligand (**2**) has the most considerable difference in IC_50_, slightly favoring Arg2 over Arg1 [171].

Recent sulfamoyl and guanidinium Cα-substituted ABH analogs, 3 and 4 respectively, have been developed, with the latter compound revealing higher inhibitory potency against Arg1 and improved pharmacokinetic profile (elimination half-life approx. 1 day) [172]. Compound 4 was also evaluated regarding its enantiomers, showing greater inhibitory potency (approx. 210 times higher) of the *R*-enantiomer over the *S*-enantiomer (Figure 5). Nonetheless, tests with the racemic mixture merely doubled the IC_50_ when compared to the optimal enantiomeric conformation (*R*-enantiomer) [172], which indicates that extra purification efforts can be skipped, at least for initial proof-of-concept purposes where inactive enantiomers do not affect parallel processes (e.g., if used in trace amounts).

The addition of piperidine, 5, or tropane, 6, substituents to the Cα of an ABH analog were shown to improve even further the inhibitory potency towards arginase, in comparison to ABH (Figure 5) [171]. This latter scaffold locks the molecular conformation of the ligand in an advantageous spatial arrangement that benefits the interaction of the N-atom of tropane directly to Asp-183 (or Asp-202), and also to Asp-181 (or Asp-200) through a bond mediated by a water molecule. The Cα substitution with 1-butyl-4-chlorophenyl or 1-butyl-2,4-dichlorophenyl groups to the tropane moiety led to the most potent Arg1 and Arg2 inhibitors of this scaffold, respectively 7 and 8. Despite the poor oral bioavailability of these piperidine/tropane scaffolds, they showed good bioavailability (>50%) when administered intraperitoneally in rodents [171].

An ABH-based ring-constrained scaffold with reduced entropy, 9, was recently developed to lock all the critical elements relevant to the inhibitory potency of the ligand in an optimal binding orientation [173] (Figure 5). The pyrrolidine nitrogen allows additional hydrogen and ionic bonding interactions, which are likely responsible for the significant increase in the inhibitory potency compared to ABH. Compound 10 became the inhibitor with the most differential specificity between isozymes, having a nearly 7-fold increased potency for Arg1 relative to Arg2 [173], whereas 11 can be regarded as the most potent arginase inhibitor synthesized to date [173].

The most recent scaffold of ABH-derived arginase inhibitors was inspired by compound 9, with the cyclopentane moiety being replaced by a rigid bicyclic center (Figure 5) [174]. The secondary amine from the pyrrolidine group of compound 12 is positioned at an ideal distance from Asp-181 (or potentially Asp-200 from Arg2) to establish an extra electrostatic interaction with the Arg1 binding site. Regardless of the modest oral bioavailability (7%) and reduced membrane permeability, compound 12 was successfully demonstrated to increase serum levels of L-arginine when administered orally in a carcinoma mouse model [174]. Substitutions in the secondary amine to improve pharmacokinetics proved to be unsuccessful. A reasonable improvement was achieved with a fluorooctahydropentalene bearing a methylamino motif, **13** [174]. From a molecular imaging point of view, it is interesting to state that, among the few fluorinated arginase inhibitors reported in the literature [171,173,174], compound **13** is the most promising. The synthesis of a radiofluorinated analog of this compound may hold great potential for positron emission tomography (PET) imaging detection of arginase-related processes.

Although the IC_50_ values reported in the literature between the arginase inhibitors cannot be straightly and uncritically compared due to the diverse methodologies used, the inhibitory potency of the scaffolds from the third generation of compounds usually remains in the nanomolar range. Due to the active-site homology, a proper subtype selectivity remains unattainable, which, in an era of precision therapy procedures, could have hindered potential therapeutic applications. However, even though Arg2 has been regarded as a more suitable therapeutic target as it would avoid undesirable effects on the liver [175], most arginase-related pathological effects appear to be associated with the combined activity of both isoforms [57]. Furthermore, the role of Arg1 is generally better understood than that of Arg2, especially in a broad spectrum of tumor microenvironments, where the depletion of L-arginine promotes the immune escape of cancer cells and inhibits the proliferation and activation of inflammatory and antitumor cytokines (type 1 helper T cells) [176,177]. Thus, some arginase inhibitors from the third generation have recently entered clinical trials.

The arginase inhibitor CB-1158 [178] (Figure 5), developed by Calithera Bioscience Inc., has been evaluated for the therapeutic blockade of immunosuppressive cells in the tumor microenvironment [179] and is currently undergoing clinical evaluation in patients with solid tumors [180]. The same biopharmaceutical company developed a novel orally dosed arginase inhibitor CB-280 (undisclosed structure), which already entered phase I trials for the treatment of cystic fibrosis [181]. Another novel Arg1 and Arg2 inhibitor, OATD-02 by OncoArendi Therapeutics SA (undisclosed structure, IC_50_ < 50 nM [182]), is also expected to enter phase I trials mid-2020 to evaluate its ability to inhibit the proliferation and immune escape of cancer cells [172]. The combination of third-generation arginase inhibitors with other immune checkpoint inhibitors in a mouse glioma model recently suggested improving therapeutic response [183], which opens new doors for the development and use of arginase inhibitors. There is, for example, a potential for the clinical synergy between PD1/PDL1 checkpoint antagonists combined with arginase inhibitors to remodel pathologically impaired M2 macrophage compartments in several tumors [184,185].

Another recent class of arginase inhibitors, still with undisclosed structure, has recently been pharmacologically evaluated for the functional efficacy to reverse allergen-induced airway narrowing in ex vivo lung sections of a guinea pig model of acute allergic asthma [186]. By showing inhibition of both Arg1 and Arg2 successfully (IC_50_: Arg1 = 7.5 to 43 µm and Arg2 = 2.8 to 17.9 µm [186]), increasing local NO^•^ production in the airways, these arginase inhibitors may have a promising role as a potential treatment of allergic asthma.

### 3.3. Non-Amino Acid-Based Arginase Inhibitors

Despite some boronohexanoic acid-derived ligands being the most potent arginase inhibitors described to date, concerns about potential toxicity, limited pharmacokinetics (fast clearance and low bioavailability), and high reactivity with carbohydrates (responsible for non-specific binding to biomembranes) led to the search for alternative compounds in natural sources [175,187,188,189]. Crude mixtures, solvent fractions, and organic or aqueous extracts from several parts of diverse ethnic medicinal plants showed inhibitory activity against arginase [190,191,192,193,194,195,196,197]. Tapering these mixtures to the individual molecular constituents led to the identification of stilbene, polyphenol, and flavonoid derivatives as the main components responsible for the inhibitory effect [198,199,200].

**Figure 6:**
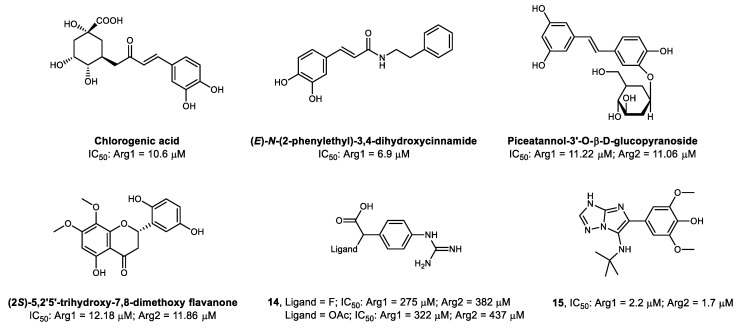
Chemical structures of non-amino acid-based arginase inhibitors.

Some of the most common plant-derived polyphenols were evaluated regarding their Arg1 inhibitory activity and compared to BEC [201]. Chlorogenic acid was identified as the most potent among the polyphenols tested (Figure 6), and its catechol moiety was found essential for the competitive inhibitory activity [201,202]. As the replacement of the ketone moiety from chlorogenic acids with a secondary amide improved the stability of the compounds, a series of cinnamide derivatives was developed against Arg1 [203]. From these derivatives, (*E*)-*N*-(2-phenylethyl)-3,4-dihydroxycinnamide showed the highest Arg1 inhibitory potency (Figure 6) and reaffirmed the importance of the catechol function [203]. The catechol group establishes hydrogen and π-π non-covalent interactions with some amino acid residues from the active-site, and both hydroxyl substituents interact with the Mn^2+^ cluster core [203]. Additionally, the reduction of the size of the carbon chain between the phenyl groups, suppression of its double bond, and substitutions in the catechol OH groups affect the inhibitory potency [203,204].

Piceatannol-3′-O-β-D-glucopyranoside, a glycoside derivative of chlorogenic acid, isolated from a rhubarb extract and tested against Arg1 and Arg2 (Figure 6), was shown to non-competitively inhibit arginase in vitro by increasing the levels of NO^•^ in isolated mice aortic rings [205]. Other experiments with piceatannol glycosides confirmed vasoprotective effects by non-competitive inhibition of arginase [206,207]. More recently, the evaluation of piceatannol analogs without the glycoside side-chain, through quantum chemistry modeling techniques, reiterated the importance of the catechol function for the binding to arginase [202]. The presence or absence of the glycoside side-chain does not seem to affect the binding affinity of piceatannol significantly, being an ideal position for the potential addition of an imaging moiety.

The isolation and screening of flavonoids from a methanol extract of the plant *Scutellaria indica* identified (2*S*)-5,2′5′-trihydroxy-7,8-dimethoxy flavanone (Figure 6) as the most potent arginase inhibitor from this specific batch [208]. The treatment of atherosclerosis-susceptible mice models with this flavonoid [145] was shown to increase the production of NO^•^ and to improve vascular function. More recently, several flavonoids have also been extracted from the leaves of Mulberry (*Morus alba*) and showed arginase IC50 values ranging from 64.1 µm to 177.2 µm [209]

Beyond the moderate IC_50_ values for arginase inhibition and the lack of isoform selectivity, all the above-mentioned plant-derived compounds have potent antioxidant activity due to the presence of a dihydroxyphenyl group in their structures, providing potential scaffolds and structural diversity for the design of new therapeutic agents targeting arginase. Furthermore, the intrinsic fluorescence emission properties in some of these natural compounds may be explored, together with optical imaging techniques, to evaluate the arginase function or expression in cell culture or tissue samples [210,211].

A new type of synthetic non-amino acid-based irreversible arginase inhibitor, **14**, was developed by going back to an approach resorting to the guanidinium function [212]. This moiety is spaced from a carboxylic acid and a leaving group by a benzene ring, which mimics the side chain length of L-arginine (Figure 6). The leaving group (preferentially an electrophile) facilitates an additional covalent interaction with a proximal nucleophilic tyrosine residue, which, together with the hydrogen bonds formed between other amino acid residues and the carboxylic acid and guanidinium functions, generates a stable complex in the active-site that permanently inhibits arginase. However, this irreversible inhibitory effect seems to be associated with modest micromolar IC_50_ values.

To date, the most potent synthetic non-amino acid-based arginase inhibitor reported is an imidazotriazole derivative containing a dimethoxyphenol function, **15** [175]. The methoxy-substituents were shown to be essential for the inhibitory activity, but due to the likely metabolic oxidation pathway converting them to benzoquinones, it may carry considerable toxicological effects.

## 4. Molecular Imaging of Arginase

The classical labeling of L-arginine with stable carbon and nitrogen isotopes (^13^C and ^15^N) allows studying the metabolic flux of this amino acid in normal or pathological tissue samples by magnetic resonance and mass spectroscopy [92]. Currently, the development of novel, highly sensitive, reliable, and reproducible methods to evaluate arginase in real time remains an active research topic [213,214,215]. These in vitro analytical methods may be useful for screening potential arginase inhibitors and assessing the extent of their inhibitory activity. However, they are not suitable to evaluate the efficiency of the inhibitors or to map the arginase expression in biological systems more complex than just cell or tissue samples, therefore failing to predict the pharmacokinetics of arginase inhibitors within living subjects. For more advanced purposes, such as aiding the pharmaceutical industry in evaluating ADME (absorption, distribution, metabolism, and excretion) properties of novel therapeutic agents targeting arginase, treatment follow-up, or even early clinical detection and diagnosis of certain arginase-related diseases, it is advantageous to use minimally invasive molecular imaging modalities. These imaging techniques can provide quantitative spatiotemporal information of biological mechanisms in vivo at the cellular and molecular level while minimally affecting the processes under study.

### 4.1. Molecular Imaging Modalities

Molecular imaging embraces a range of techniques and methodologies with the primary purpose of detecting, preferably, molecular changes in the genesis of pathophysiological disorders at an early stage. Each modality of clinical and preclinical molecular imaging has its strengths and weaknesses in terms of resolution, sensitivity, or applicability (Table 2).

Optical imaging is used in preclinical studies or clinically during guided intraoperative procedures. Due to the limited tissue penetration of the emitted light, the external detection of the probe can be challenging [216]. The use of photoluminescent gold nanoparticles coated with L-arginine exhibited efficacy to monitor arginase activity, as there is a decrease of the photoluminescence signal in the presence of this enzyme due to the hydrolysis of L-arginine from the coating [217]. As a proof-of-concept, this imaging agent was tested in rats with and without triptolide-induced liver injury and showed great promise for arginase-targeted biomedical applications [218]. However, being vectorized by L-arginine, this probe is also subject to NOS activity and may lack specificity to arginase. An interesting alternative could be the coating of these nanoparticles with an arginase inhibitor instead of L-arginine.

A different approach also using optical imaging is the intravenous injection of engineered macrophages, which release luciferase (an enzyme that produces bioluminescence) when polarized to the M2 arginase-expressing phenotype in the tumor microenvironment, enabling bioluminescence imaging and blood measurements [219].

Photoacoustic tomography holds great potential for preclinical assays, despite still being in an ascending phase of development and lacking specific probes for subcellular mechanisms [220]. The shortage of sensitive and specific probes is also delaying the application of ultrasonography as a proper molecular imaging technique for enzymatic activity, being mainly used for morphological evaluations [221,222]. Thus, the use of these techniques to detect arginase is still far away.

Since magnetic resonance imaging (MRI) has a low detection sensitivity, which affects the efficiency to quantify molecular processes, and no specific probes for arginase have been developed, the applications of MRI have been limited to the evaluation of late morphological alterations associated with arginase disorders [82,223,224]. However, there has been some exciting progress in the development of sensitive and activatable paramagnetic probes for the detection of enzymatic activity with MRI [221,222,225]. The typical need for a bifunctional chelator holding the paramagnetic contrast agent (e.g., Gd^3+^ or Fe^3+^), which serves as a signaling antenna linked to the targeting molecule through a spacer group to minimize the influence of this bulky moiety in the binding affinity, usually makes MRI not very suitable for small vectors, as is the case of the arginase ligands. Nevertheless, the chelation of paramagnetic contrast agents, together with radiometals (Table 3), may be used for the development of multimodal probes based on nanoparticles or oligomers with multiple conjugations of arginase inhibitors.

Despite not being exactly considered a molecular imaging modality, computed tomography (CT) is often combined with single-photon emission computed tomography (SPECT) or PET to allow anatomical correlation and improve the spatial resolution of these highly sensitive nuclear imaging techniques based on the detection of the decay from radioactive isotopes. Thus, due to the total body penetrance, SPECT and PET are the most suitable techniques to potentially map and evaluate the arginase expression in vivo and can provide crucial spatiotemporal information. The measurement of the radioactivity concentration and uptake in target regions can be translated into quantitative values representative of each tissue function. Regions of interest or three-dimensional volumes of interest can be drawn when using dynamic imaging protocols to create time–activity curves and to calculate standardized uptake values. For this, it is essential to radiolabel suitable molecules (radiotracers), specifically directed to the desired target and binding with high affinity, with an appropriate γ or β^+^ emitter (Table 3) without significantly affecting the active pharmacophore structure and biological activity. The sufficiency of nano- or even picomolar concentrations of a radiotracer, therefore avoiding pharmacological or toxicological side-effects, makes nuclear imaging techniques ideal for studying biological processes without affecting them.

### 4.2. Development of Arginase-Targeted Radiotracers for Nuclear Imaging

**Figure 7:**
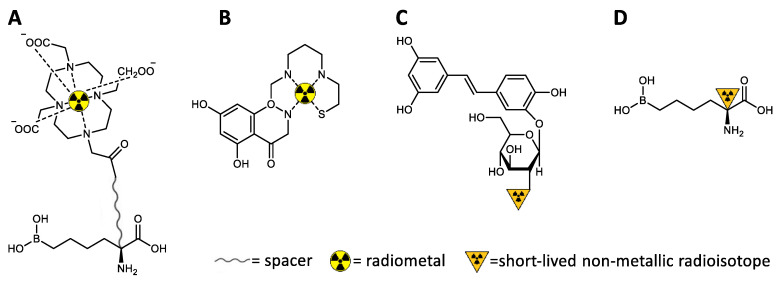
Potential radiolabeling approaches for the development of nuclear imaging radiotracers (illustrated with theoretical examples of potential arginase-targeted vectors). (**A**) Conventional chelation of radiometals (the appropriate chelator needs to be chosen for the selected radiometal; usually not suitable for small molecules targeting subcellular processes). (**B**) Integration of the radiometal in the vector structure (more challenging design; may be applied to more intricate scaffolds, e.g., to mimic flavonoids). (**C**) Radiolabeling of prosthetic groups and further conjugation to the central vector (potential strategy to radiolabel non-amino acid-based arginase inhibitors, e.g., piceatannol glucopyranoside analogs). (**D**) Direct radiolabeling of the molecular vector causing minimum structural disturbances (potentially useful to radiolabel Cα-substituted ABH analogs).

To date, there are no specific radiotracers targeting arginase. On the other hand, a few radiotracers have been developed and directed to NOS [226,227,228,229,230,231,232,233]. Regardless of the technical characteristics of SPECT and PET, the preferred sphere of action of these radiotracers falls into the latter tomographic modality, mainly because small molecules can be better labeled with carbon-11 or fluorine-18 as usually less structural changes are needed. Similar to what happens for MRI contrasts, there is often a structural inadequacy for radiometals to be incorporated into small molecules without interfering much with the pharmacophore region, polarity, and binding affinity. The need for a bulky bifunctional chelator to link the radiometal to the vector molecule may hinder the capacity of the radiotracer to diffuse and cross membranes, to adopt an optimal orientation towards the binding site, and to establish the appropriate interactions with the active enzyme residues [233]. Thus, since NOS and arginase have in common the same physiological substrate, similar considerations apply to the development of new radiotracers targeting arginase. Besides, PET has higher sensitivity, requires lower doses of radioactivity, and enables more accurate quantitative analysis than SPECT, being especially suitable for mapping subcellular processes with small radiolabeled molecules (Figure 7) [221].

The synthesis of L-arginine analogs, substituting intrinsic C, N, or H with their isotopes (^13/14^C, ^15^N, or ^3^H), is an old technique that remains valid to evaluate arginase activity, as both the substrate and the resulting products (urea and L-ornithine) can be quantified through diverse analytical techniques [234,235,236,237,238,239]. However, a similar strategy using ^13^N or ^11^C to synthesize an L-arginine analog to be used as PET radiotracer is not a viable option for mapping the in vivo arginase expression and activity. First, L-arginine is not specific for arginase, as it is also a NOS substrate. More importantly, a hypothetical [^13^N/^11^C]arginine radiotracer would follow the L-arginine natural metabolic pathway, being hydrolyzed to urea, L-ornithine, L-citrulline, and NO^•^, which would also be radioactive (depending on the positions radiolabeled in L-arginine) and would follow their specific biological and clearance paths. This cascade of metabolic processes may create a high background of radioactive signals hardly possible to be isolated, distinguished, and understood through in vivo real-time imaging [236]. Furthermore, radioactive analogs of natural amino acids need to be labeled with radioisotopes of their fundamental elements, i.e., ^13^N and ^11^C, having the inherent limitation of a short physical half-life (approx. 10 and 20 min, respectively).

**Figure 8:**
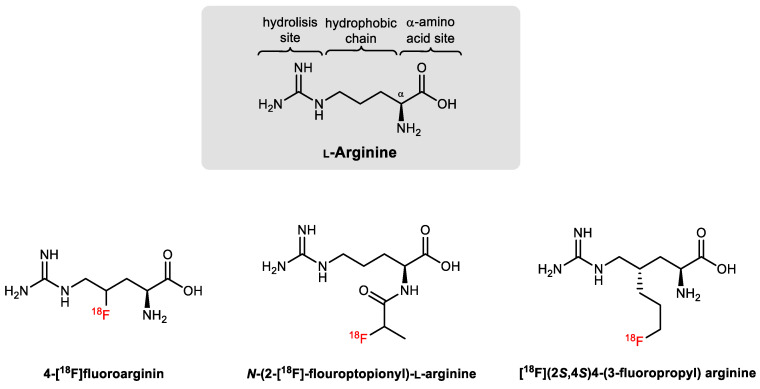
Reported examples of ^18^F-labeled L-arginine derivatives [243,244,245].

The time constraints imposed by ^13^N and ^11^C are not always compatible with the entire process of making batch productions of a PET radiotracer for intravenous injection, in vivo study of the metabolic pathway, biodistribution, and analysis of metabolites. Thus, although fluorine is not present in natural amino acids, the longer physical half-life of ^18^F and the greatest sterical and electronic similarities to hydrogen (which enable the conservation of bond length, strength, and atomic radius when substituting a C-H or C-OH group for a C-F), made this radioisotope very desirable for the radiolabeling of amino acid derivatives [240,241,242]. The addition of fluorine can, however, modify the lipophilicity, pKa, and the biological properties of natural substrates, especially in small molecules such as L-arginine (Figure 8), since slight structural changes or the addition of prosthetic groups are needed to accommodate ^18^F [243,244,245].

Although they were not designed or evaluated to specifically or selectively bind arginase, the ^18^F-labeled arginine derivatives reported in the literature showed good potential for PET imaging of the arginine metabolism in tumors [243,244,245], especially if compared with the image pattern theoretically expected for the natural substrate analog [^13^N/^11^C]arginine [236]. If cleverly designed, the advantage of using radiolabeled modified amino acids, from a PET-imaging point of view, is that they do not metabolize in the same way as the natural ones [241]. Ideally, to image arginase, an L-arginine derived radiotracer should be efficiently processed by the enzyme (low *K_m_*), producing a radiolabeled metabolite that becomes trapped in the cells or tissues expressing arginase. This selective accumulation will amplify the radioactive signal, correlating the uptake with the arginase activity or expression. Meanwhile, the not-metabolized fraction of the radiotracer and the non-targeted metabolites should be cleared and excreted, enhancing the target-to-background ratio in the PET image of arginase [241].

In contrast to paramagnetic or optical probes, nuclear imaging radiotracers cannot be synthesized to have an activatable “switch on/switch off” type of signal to improve the target-to-background ratio. Thus, the amplification of the emitted radioactive signal and the enhancement of the contrast between arginase-expressing and -non-expressing regions has to be achieved by using appropriate radiochemistry approaches to synthesize specific and selective radiotracers in high purity and molar activities [246,247]. The development of radiotracers that efficiently bind to arginase with high affinity and specificity is, therefore, essential.

Although the pharmacokinetics and toxicity profiles of the latest generations of highly potent arginase inhibitors (Figure 5) are not always suitable for further therapeutic applications, these molecules have a promising potential to be revived as PET radiotracers. The high binding affinities shown by these inhibitors, the advantage of their fast clearance to potentially enhance target-to-background contrast, and the nano- to picomolar concentration range needed for PET, make ABH-derived arginase inhibitors very promising radiotracers for imaging. The reversible binding nature of boronic acid-containing arginase inhibitors makes them ideal for mapping arginase expression, as these ligands bind in a 1:1 ratio with the enzyme regardless of catalytic activity, accumulating preferentially in areas of high concentration of the enzyme protein [241]. On the other hand, due to the rapid elimination half-life, these inhibitors will diffuse away from tissues with low arginase concentration. Furthermore, the generally well-tolerated substitutions at Cα indicate an ideal site for the introduction of nuclear imaging agents without significantly affecting the biological activity.

### 4.3. Future Perspectives for Arginase-Directed Radiotracers

Currently, none of the identified arginase inhibitors has shown pharmacologically significant selectivity to one of the arginase isoforms over the other. Achieving this selectivity would be very useful to understand the role of arginase isoforms in normal and pathological metabolism. However, the arginase-related pathological effects are often associated with the activity of both isoforms [57]. Thus, despite the low selectivity for the isoforms, a few compounds from the third generation of arginase inhibitors have recently entered clinical trials (e.g., CB-1158, CB-280, and OATD-02) to assess efficacy in the treatment of fibrosis or immunosuppressive tumors. The evaluation of these compounds would probably be accelerated if a β^+^-emitter radiolabeled analog was available, as it would benefit from real-time pharmacokinetic monitoring (e.g., by competition studies against their ^11^C-labeled analogs). Nevertheless, it is not always possible to technically or efficiently achieve an exact radiolabeled analog of the arginase inhibitor without having to modify it slightly, which would alter the final biological activity and kinetics. For example, a ^11^C-radiolabeled analog of CB-1158 may be achievable but would require exceptional levels of expertise, especially due to the limited physical half-life of ^11^C.

The availability of a reference radiolabeled arginase inhibitor may be useful to evaluate the therapeutic efficiency of novel arginase inhibitors by real-time in vivo competitive studies, or by follow-up of the disease progression after a treatment cycle. Although the arginase inhibitors were not always efficient for the pharmacological purpose for which they were synthesized, due to the toxicity profile or the limited pharmacokinetics (fast clearance and low bioavailability), the third generation of highly potent arginase inhibitors contains interesting lead compounds for the development of radiotracers targeting arginase. For instance, the presence of a chlorophenyl group in compound **1** (IC_50_: Arg1 = 330 nM and Arg2 = 1120 nM [171]) and **7** (IC_50_: Arg1 = 17 nM and Arg2 = 30 nM [171]) may open perspectives for the development of [^18^F]fluorophenyl analogs via several late-stage ^18^F-fluorination options [248]. Compound **13** (IC_50_: Arg1 = 6 nM [174]), which contains a fluorocyclopentane moiety in its structure, may also serve as a lead for the development of a radiolabeled analog.

Despite generally having lower inhibitory potency than the synthetic substrates, non-amino acid-based arginase inhibitors derived from plant extracts (Figure 6) may also be attractive scaffolds for radiolabeling. The heteroaromatic structure of flavonoids may allow more innovative strategies, such as the synthesis of flavonoid-mimicking compounds integrating a chelator-like core for further conjugation to a radiometal (Figure 7B) [249]. However, a more reliable approach may be the labeling of flavonoids via late-stage aromatic ^18^F-fluorination strategies, which should lead to less structural changes affecting the bioactivity. The use of [^18^F]fluorodeoxyglucose ([^18^F]FDG)-based prosthetic groups [250] may also be an interesting approach (Figure 7C) to synthesize radiofluorinated analogs of the piceatannol glucopyranoside (Figure 7, IC_50_: Arg1 = 11.22 mm and Arg2 = 11.06 mm [205]). A ^11^C-labeled analog of compound **15**, the most potent non-amino acid-based arginase inhibitor reported (IC_50_: Arg1 = 2.2 µm and Arg2 = 1.7 µm [175]) may also be synthesized by a conventional reaction with [^11^C]CH_3_I [251]. Despite the concerns about the possible toxicity of this ligand when administered at therapeutical doses, the picomolar concentrations typically needed for PET imaging would enable its use with a minimum risk of side effects. However, the moderate IC_50_ values of the non-amino acid-based arginase inhibitors may be insufficient for successful PET imaging.

A different approach to be explored is the radiolabeling of commercially available anti-arginase antibodies [252]. This radiolabeling may be performed either by the conjugation of radiometals using bifunctional chelating agents, which might even enable the development of multimodal imaging probes, or by the radiolabeling of the antibody fragments with radiofluorinated prosthetic groups [253].

In summary, the radiolabeling of the most promising ABH-derived arginase inhibitors holds high potential for diagnostic and research applications. Beyond mapping changes in arginase expression, thereby detecting potential pathological processes (Table 1) at early stages, these radiotracers may also aid the pharmaceutical industry in assessing the target engagement, biodistribution, and pharmacokinetics of the arginase inhibitors in real-time. During clinical trials, follow up PET scans using the radiolabeled arginase inhibitors can support evaluation of the efficacy of therapeutic cycles with the non-radioactive analogs or other novel arginase inhibitors, helping to determine the required dose for significant in vivo inhibition of the enzyme. Ultimately, these radiotracers may also be used to select those patients who can benefit the most from treatments with arginase inhibitors.

## 5. Conclusions

The development of arginase inhibitors by structure-based drug design resulted in several potent compounds. Nevertheless, the high inhibitory potencies usually revealed in vitro are not always matched by a suitable in vivo stability, bioavailability, or pharmacokinetic profile, such as a biological half-life or target residence time sufficiently extended to enhance the therapeutic effect. Therefore, there are currently no arginase inhibitors available for clinical use, and only a few compounds have made it to the first phases of clinical trials. Additionally, there is still an unmet need for arginase inhibitors with pharmacologically significant selectivity to each arginase isoform, which would be useful to understand the exact role of the enzyme subtypes in certain pathologies and to establish the advantages (if any) above non-isozyme-selective inhibitors. Thus, the synthesis of novel and enhanced arginase inhibitors remains a very active research field. Therefore, these arginase inhibitors may have a second life outside therapeutic applications by being used as reference molecules for the development of molecular imaging probes.

Therefore, this review aimed to explore the potential of arginase as an imaging biomarker and to stimulate interest in the development of increasingly specific and selective arginase-targeted imaging probes. These imaging probes may become an essential clinical and research tool to estimate the effective arginase concentration in some of the most prominent arginase-expressing pathologies (e.g., fibrotic conditions, atherosclerosis, asthma, immunosuppressive tumors, or carcinomas). Nevertheless, a persistent drawback is the lack of subtype-selective arginase inhibitors, which are challenging to design due to the minor structural variations between the isozyme active-sites. Achieving subtype selectivity would be essential to understand if polymorphisms of Arg1 and Arg2 are associated with disease severity, poor prognosis, or reduced responsiveness to therapeutic approaches. Hence, finding subtype-selective arginase inhibitors remains the ultimate goal to be achieved in this field.

## Figures and Tables

**Table 1:** Arginase-dependent pathological conditions, proposed trigger signals, and mechanisms.

Pathology	Animal/Cell Line Model	Arginase Levels	Proposed Trigger Signal	Proposed Disease Mechanism	Ref.
Diabetes-induced vasculo-pathy	Bovine aortic endothelial cells exposed to glucose or activated for Arg1 upregulation by adenoviral delivery; Arg1-deficient mouse model	↑ Arg1	Glucose treatment activates Rho-associated protein kinases, which induce macrophages to upregulate Arg1	Substrate depletion by Arg1 reduces NO^•^ and leads to impaired vascular relaxation, increased blood flow, and upsurge of reactive oxygen species, which causes premature endothelial cell senescence and defective vascular repair	[58]
	Diabetic mouse model; blood samples from diabetic patients		Increased plasma glucose levels induce the release of Arg1 via serum exosomes		[59]
	Mice induced to diabetes by streptozotocin; bovine retinal endothelial cells		High glucose levels activate NOX2 leading to upregulated Arg1		[60]
Obesity-induced vasculo-pathy	Diet-induced obesity and metabolic syndrome mouse model	↑ Arg1	High-fat, high-sucrose treatment activates Rho-associated protein kinases, which increases Arg1 expression	Upregulated synthesis of polyamines by Arg1 promotes cell proliferation and fibrosis; increased levels of reactive oxygen species contribute to dysfunction	[61]
Arterial thickening, fibrosis, and stiffening	Arg1-deficient mouse model; rat aortic smooth muscle cells	↑ Arg1	Angiotensin II acts upon the renin–angiotensin system and induces arginase upregulation	Enhanced synthesis of polyamines/proline leads to vascular cell proliferation and collagen formation, which changes smooth muscle tone	[62]
Hyper-tension	Obese and lean male rat models	↑ Arginase^1^	Obesity-induced arginase upregulation	L-Arginine depletion reduces NO^•^-mediated arterial vasodilation	[63]
Arterio-genesis	Male mice submitted to peripheral arteriogenesis; mouse primary artery endothelial cells and smooth muscle cells	↑ Arg1	Shear stress induces monocytes maturation to macrophages, which impairs M1/M2 to favor Arg1 expression	Enhanced Arg1 activity promotes perivascular M2 macrophage accumulation, which contributes to cell proliferation	[64]
Myocardial infarction	Male mouse submitted to surgical ligation of the left anterior descending coronary artery to induce myocardial infarction	↑ Arg1	Neutrophils are recruited and infiltrate into the infarcted area, activating the macrophages to favor Arg1 expression	Increased Arg1 activity results in enhanced proline and collagen synthesis, leading to fibrosis, ventricular remodeling, and eventual heart failure	[65]
Erectile dysfunction	Patients with a medical diagnosis of erectile dysfunction	↑ Arg1 and Arg2	Genetic polymorphisms induce Arg1 and Arg2 expression and activity	L-Arginine depletion leads to endothelial dysfunction and impaired smooth muscle relaxation; erectile dysfunction is an early sign of cardiovascular diseases	[66]
Chronic obstructive pulmonary disease	Ex vivo pulmonary vascular tissue from smokers	↑ Arg1	Tobacco smoking upregulates the arginase pathway	Imbalance of polyamines/NO^•^ causes vascular remodeling, airway dysfunction, and oxidative stress	[67]
Pulmonary hypertension	Human pulmonary artery smooth muscle cell	↑ Arg2	Induced hypoxia activates protein kinases and transcription factors leading to the upregulation of Arg2 expression	Increased synthesis of polyamines leads to vascular smooth muscle cell proliferation and remodeling; decreased NO^•^ synthesis impairs vasodilation, which contributes to dysfunction and pulmonary hypertension	[68]
	Human pulmonary artery smooth muscle cell; male mice exposed to hypoxia				[69]
Pulmonary fibrosis	Male mice with bleomycin-induced pulmonary fibrosis	↑ Arg2	Pro-inflammatory T helper cells change M1/M2 polarization and increase Arg2 expression	Increased biosynthesis of polyamines and collagen activates lung fibroblast proliferation and differentiation	[70]
	Primary bronchial cultures from cystic fibrosis patients	↑ Arginase^1^	F508del gene mutation leads to excessive arginase activity in the pulmonary tissue	Increased arginase expression results in a build-up of fibrotic mass; a decrease of NO^•^ levels induces the deregulation of epithelial fluid transport in the lungs and reduce cilia motility	[71]
	Cystic fibrosis pediatric patients			High levels of arginase promote collagen deposition and NOS uncoupling, causing oxidative stress and tissue damage	[72]
	Cystic fibrosis patients	↑ Arg1	Recessive gene mutation leads to an excessive arginase activity in pulmonary tissue	Reduced NO^•^ impairs smooth muscle relaxation, bronchodilation, and bacterial killing mechanisms	[73]
Asthma	Asthmatic patients	↑ Arg1	Allergen activation of IgE leads to neutrophil infiltration in lungs and activation of M2 arginase-expressing macrophages	Upregulation of Arg1 increases mucus production and smooth muscle contraction. Arg1 seems to correlate to bronchial asthma	[74]
		↑ Arg2	Chronic airway inflammations have high co-expression of Arg2 and iNOS	Arg2 delivers L-ornithine into mitochondria, providing nitrogen to an autonomous L-arginine-NO^•^-citrulline cycle and sustaining high NO^•^ levels, which seems related to more severe and reactive conditions	[75]
	Human bronchial epithelial cell line (BET1A); Arg2-deficient mice with allergen-induced asthma	↑ Arg2	Allergens enhance hypoxia-induced factors, which activate IL-13 to upregulate Arg2	Increased Arg2 is suggested to be a counter-regulatory mechanism to reduce signal transduction and suppress airway inflammation	[76]
	Mite-challenged NC/Nga mouse model of asthma	↑ Arg1	Allergen activation induces the expression of arginase-upregulating mechanisms	Arginase decreases NO^•^ levels, suppressing anti-inflammatory, bronchodilatory, and vascular modulating effects	[77]
Chronic rhino-sinusitis	Fragments of mucosa collected from the ethmoid sinus of chronic rhinosinusitis patients	↑ Arg2	Several cytokines found in the sinus mucosa lead to enhanced arginase expression	Increased Arg2 leads to cell and collagen proliferation and decreases NO^•^ levels, which suppresses bronchodilatory and anti-inflammatory effects	[78]
Tuberculosis	Tissue samples from active tuberculosis patients; mouse model infected with *Mycobacterium tuberculosis*	↑ Arg1	Intracellular parasites circumvent NO^•^ toxicity through the induction of Arg1-expressing macrophages in lungs	High Arg1 expression leads to collagen deposition and lung damage, which drives to inflammation by inhibiting type 1 helper T cells	[79]
Inflammatory bowel disease	Mouse model of inflammatory bowel disease by dextran sulfate sodium induction	↓ Arg1	Extracellular matrix protein 1 (ECM1) in macrophages impairs M1/M2 polarization decreasing the expression of Arg1	Reduction of Arg1 suppresses tissue repair mechanisms and, together with upregulated expression of inflammatory cytokines, increases chronic inflammatory response	[80]
Autoimmune (type 1) diabetes	Diabetic female mouse model induced by hyperglycemia	↑ Arg1	Increased plasma glucose levels impair M1/M2 polarization	Decreased NO^•^ levels lead to a pro-inflammatory effect, weakening innate immunity	[81]
Arthritis	Synovial tissue samples from rheumatoid arthritis patients; arthritis mouse model (K/BxN)	↓ Arg1	Transcription factor Fos-related antigen 1 downregulate Arg1 expression by binding to the promoter region	Reduction of Arg1 suppresses polyamines synthesis and subsequently downregulates tissue repair mechanisms and counter-regulates pro-inflammatory cytokines	[82]
Multiple sclerosis	Arg2-knockout mice with induced autoimmune encephalomyelitis	↑ Arg2	Impaired M1/M2 macrophage polarization	Upregulated Arg2 stimulates the production of T helper 17 cells-differentiating cytokines, which induces inflammation	[83]
Viral infection	Patients with severe fever and thrombocytopenia syndrome	↑ Arg1	Viral-induced impairment of M1/M2 polarization favors the upregulation of Arg1	Arg1 causes L-arginine deficiency, which is associated with decreased NO^•^ and suppresses antiviral immunity	[84]
	Mice infected with *Trypanosoma cruzi* and *Schistosoma mansoni*				[85]
	Peripheral lymph node cells from HIV patients				[86]
Peritonitis	Murine macrophage-like cell line (RAW264.7) and human monocyte cell line (THP-1)	↑ Arg1	IL-4-stimulated inflammation upregulates cytochrome P450 1A1, which impairs M1/M2 polarization	Increased Arg1 expression is associated with compensatory response mechanisms against an uncontrolled inflammation	[87]
Acute myeloid leukemia	Human acute myeloid leukemia cell lines (THP-1, U937, MOLM16, K562)	↑ Arg2	Increased acute myeloid leukemia blast cells overexpressing Arg2	Arg2 activity reduces IFN-γ and inhibits T cell immune-suppressive response	[88]
Chronic myelo-monocytic leukemia	Human bone marrow mononuclear cells	↑ Arg1	Mutations in epigenetic regulators upregulate Arg1	L-Arginine depletion by Arg1 suppress T-cells and contributes to immune evasion	[89]
Basal-like breast cancer	Human mammary epithelial cells (HeLa, HMEC, HMEC-ras, MDA-MB-231, MDA-MB-468)	↑ Arg2	Oncogene transformations trigger Arg2 expression	Arg2 upregulated between DNA synthesis and mitotic phases of cancer cells cycle promotes cell proliferation	[90]
Neuro-blastoma	Neural crest cell line (R1113T); neuroblastoma cell lines (SKNAS, KELLY, LAN-1, IMR-32,); Ewing’s sarcoma cell line (SKNMC); sympathetic ganglion-derived stem cells (SZ16)	↑ Arg2	IL-1β and TNF-α established a feedback loop to upregulate Arg2 expression via p38 and extracellular regulated kinases signaling	Arg2 induces cell proliferation and an immunosuppressive microenvironment due to inhibition of T cell cytotoxicity	[91]
Pancreatic ductal adeno-carcinoma	Human pancreatic ductal adenocarcinoma cell lines (AsPC-1, HPAC, MIA PaCa-2, PANC-1, SUIT-2, PA-TU-8988T); Arg2-deficient mouse pancreatic ductal adenocarcinoma cell lines	↑ Arg2	Arg2 is increased upon obesity and as a result of activating oncogenic mutations	Tumors (but not cultured cancer cells lacking the in vivo tumor microenvironment) need arginase to dispose of the excess of nitrogen accumulated to enhance tumorigenicity	[92]
Melanoma	Patient with metastatic L-arginine auxotrophic melanoma	↑ Arg2	Defects in the expression of OTC and ASS enzymes result in a dependence of extracellular L-arginine; counter-regulatory mechanisms lead to the upregulation of Arg2	Tumor cells were shown to be auxotrophic and avid for L-arginine to keep cell proliferation; high expression of Arg2 is induced to increase catalytic efficiency	[93]
	Human melanoma cell lines from patients with melanoma metastasis adhered to confluent human umbilical vein endothelial cells layers		Pro-inflammatory T helper cells change M1/M2 polarization and increase Arg2 expression	Arg2 enhances melanoma cell proliferation through polyamine production and promotes metastasis through enhancing H_2_O_2_ production and STAT3 signaling	[94]
Ovarian carcinoma	Human ovarian cancer cell lines (OVP-10, AD-10, A2780, Skov3, CaOv-3, MDAH2774, OvCa-14)	↑ Arg1	Tumor-derived exosomes containing Arg1 are released into circulation	Increased Arg1 expression inhibits antigen-specific T-cell proliferation and is related to a worse prognosis	[95]
Osteosarcoma	Human osteosarcoma cell lines (SaOS-2 and OS-17)	↑ Arg2	Hypoxic environment upregulates Arg2	Arg2 induces immunosuppression by inhibition of T-cells function	[96]
Glioma	Mouse glioma cell lines (GL261, KR158B)	↑ Arg1	Myeloid-derived suppressor cells overexpressing Arg1 infiltrate into the tumor	Increased Arg1 expression suppresses the efficacy of the immune system	[97]
Hepato-cellular carcinoma	Human hepatocellular carcinoma cell line (Huh7)	↑ Arg1	Impaired M1/M2 polarization induces Arg1 upregulation	Overexpression of Arg1 promotes cell proliferation, migration, and invasion, being a critical process in cancer metastasis and progression	[98]
	Patients with advanced hepatocellular carcinoma		Deprivation of L-arginine recycling enzymes OTC and ASS at the transcription or translational level	Tumor auxotrophic for L-arginine to enable cell proliferation and viability; L-arginine deprivation therapy can be a therapeutic approach	[99]
Cervical cancer	Human squamous cell carcinoma cells from patients	↑ Arginase^1^	Increased levels of circulating IL-10 and decreased levels of IFN-γ enhance arginase activity	Upregulated arginase levels contribute to the tumor immunosuppressive microenvironment	[100]
Alzheimer	Alzheimer’s disease mouse models	↑ Arg1 and Arg2	Microglial activation results in cytokines production, which induces the expression of arginase in brain	Arginase overexpression at β-amyloid deposition sites leads to NOS uncoupling, O_2_^•−^ generation, and neuro-degenerative oxidative stress	[101]
Acute traumatic brain injury	Male rats submitted to traumatic brain injury surgery	↑ Arg1	Elevation of pro-inflammatory cytokines induces Arg1 expression	Increased Arg1 leads to eNOS uncoupling and enhances oxidative stress, inflammation, and vascular dysfunction	[102]
Fronto-temporal dementia	Male transgenic mice expressing a mutant form of human microtubule-associated protein tau	↑ Arginase^1^	Mutations in microtubule-associated protein tau	Functional significance of arginase remains uncertain as the production of polyamines enhances microtubule stability, which should reduce inflammation and tau proteins	[103]
Neuro-degeneration and neuro-vascular permeability	Male mice treated with homocysteine to induce vascular dysfunction and stroke-like symptoms	↓ Arginase^1^	Elevated levels of homocysteine, produced from methionine, lead to hyperhomocysteinemia, impairing NOS pathway	Upregulated NO^•^ levels lead to nitrosative stress, extracellular matrix degradation, blood–brain barrier permeability, and neurodegeneration	[104]
Huntington’s disease	Post-mortem brain sections from patients with Huntington’s disease	↑ Arg1	Metabolic impairment of the urea cycle in the brain	Increased urea in the brain induces neurodegeneration by impaired osmoregulation	[105]
Acute ischemic stroke	Peripheral blood samples from patients with a first-ever acute ischemic stroke	↑ Arg1	Stroke induces the downregulation of a microRNA, which upregulates the Arg1 expression	Increased Arg1 acts against the activation of pro-inflammatory signals after stroke but may also be implicated in stroke-induced immunosuppression	[106]
Cerebral ischemia and excitotoxicity	Arg2-knockout mice with permanent distal middle cerebral artery occlusion or induced excitotoxicity	↓ Arg2	Arg2 deficiency worsens brain injury after an ischemic event	Arg2 may play a substantial protective role by regulating NO^•^ levels and controlling reactive species	[107]

↑ increased levels; ↓ decreased levels; ^1^ unspecified arginase isoform.

**Table 2:** Properties of the molecular imaging tomographic modalities.

Technique	Imaging Agent	Spatial Resolution	Detection Sensitivity	Penetration Depth	Quantification Efficiency
Optical	Fluorophores or lanthanides	2–3 mm	10^−11^ mol·L^−1^	<20 mm	Medium
Photoacoustic	Light absorbing agents	0.1–1 mm	10^−11^ mol·L^−1^	<70 mm	Medium
Ultrasound	Gas microbubbles	0.5–1 mm	10^−8^ mol·L^−1^	<200 mm	Low
MRI	(Super)para-magnetic agents	0.03–1 mm	10^−5^ mol·L^−1^	>300 mm	Medium
CT	I or Ba agents	0.03–1 mm	0.1 mol·L^−1^	>300 mm	−
PET	β^+^-emitters	1–10 mm	10^−12^ mol·L^−1^	>300 mm	High
SPECT	γ-emitters	0.5–15 mm	10^−11^ mol·L^−1^	>300 mm	High

**Table 3:** Features of some of the most commonly used imaging radionuclides.

Modality	Radio-nuclide	Physical Half-Life	Production	Target /Parent Isotope	Primary Precursor	Main Emissions
SPECT	^99m^Tc	6.01 h	Generator (^99^Mo/^99m^Tc)	^99^Mo, parent isotope	[^99m^Tc]TcO_4_Na	γ, 141 keV
	^111^In	2. 81 d	Cyclotron ^111^Cd(p,n)^111^In ^112^Cd(p,2n)^111^In	^111/112^Cd-enriched sample	[^111^In]InCl	γ, 245, 171 keV
	^67^Ga	78.3 h	Cyclotron (^68^Zn(p, 2n)^67^Ga)	^68^Zn-enriched sample	[^67^Ga]GaCl_3_	γ, 300, 181, 93 keV
	^123^I	13.2 h	Cyclotron (^124^Te(p, 2n)^123^I)	^124^Te-enriched sample	[^123^I]I_2_	γ, 159 keV
PET	^13^N	9.97 min	Cyclotron ^16^O(p, α)^13^N	H_2_O H_2_O+ethanol	[^13^N]NO_2/3_ [^13^N]NH_3_	100% β^+^, 1200 keV
	^11^C	20.4 min	Cyclotron ^14^N(p,α)^11^C	N_2_(+O_2_) N_2_(+H_2_)	[^11^C]CO_2_ [^11^C]CH_4_	99% β^+^, 960 keV
	^18^F	109.8 min	Cyclotron ^20^Ne(d,α)^18^F ^18^O(p,n)^18^F	Ne(+F_2_) [^18^O]H_2_O	[^18^F]F_2_ [^18^F]F^−^ aq.	97% β^+^, 630 keV; 3% electron capture
	^68^Ga	68 min	Generator (^68^Ge/^68^Ga)	^68^Ge, parent isotope	[^68^Ga]GaCl_3_	90% β^+^, 1830 keV; 10% electron capture
	^64^Cu	12.7 h	Cyclotron ^64^Ni(p,n)^64^Cu	^64^Ni-enriched sample	[^64^Cu]CuCl_2_	18% β^+^, 650 keV; 39% β^−^, 579 keV; 43% electron capture
	^89^Zr	78.4 h	Cyclotron ^89^Y(p,n)^89^Zr	^89^Y-enriched sample	[^89^Zr]Zr(C_2_O_4_)_2_	77% electron capture; 23% β^+^, 902 keV

## Abbreviations

ABH	2-(*S*)-Amino-6-boronohexanoic acid
Arg1	Arginase type I
Arg2	Arginase type II
ASS	Argininosuccinate synthase
BEC	*S*-(2-Boronoethyl)-L-cysteine
cAMP	Cyclic adenosine monophosphates
COX	Cyclooxygenase
CT	Computed tomography
FABH	2-Amino-6-borono-2-(difluoromethyl)hexanoic acid
IC_50_	Half-maximal inhibitory concentration
IFN	Interferon
IgE	Immunoglobulin E
IL	Interleukin
*k_i_*	Inhibitory constant
*K_m_*	Michaelis-Menten kinetics
MABH	2-Amino-6-borono-2-methylhexanoic acid
MRI	Magnetic resonance imaging
NOHA	*N*^ω^-hydroxy-L-arginine
NOS	Nitric oxide synthase
NOX	Nicotinamide adenine dinucleotide phosphate oxidase
OTC	Ornithine carbamoyltransferase
PET	Positron emission tomography
SPECT	Single-photon emission computed tomography
STAT3	Signal transducer and activator of transcription 3
TGF	Transforming growth factor
TNF	Tumor necrosis factor
*V_max_*	Maximum rate of reaction
